# Infratuberosity Anterior Closing-Wedge High Tibial Osteotomy for Slope Correction in Anterior Cruciate Ligament–Deficient Knees

**DOI:** 10.1016/j.eats.2024.103153

**Published:** 2024-07-27

**Authors:** Matthieu Ollivier, Wiemi Douoguih, Karam Mark Karam, Shintaro Onishi, Te-Feng Arthur Chou

**Affiliations:** a Sainte-Marguerite Hospital, Institute for Locomotion, Department of Orthopedics and Traumatology, Aix-Marseille University, AP-HM, CNRS, ISM, Marseille, France; b Department of Orthopedics and Traumatology, Institute of Movement and Locomotion, St. Marguerite Hospital, Marseille, France; c MedStar Lafayette Orthopaedic and Sports Medicine Center, MedStar Washington Hospital Center, Washington, District of Columbia, U.S.A.; d Department of Orthopaedic Surgery, MedStar Georgetown University Hospital, Washington, District of Columbia, U.S.A.; e Georgetown University School of Medicine, Washington, District of Columbia, U.S.A.; f Department of Orthopaedic Surgery, MedStar Union Memorial Hospital, Baltimore, Maryland, U.S.A.

## Abstract

Graft failure after anterior cruciate ligament reconstruction is multifactorial, with increased tibia slope identified as one of the risk factors. Several slope-correcting osteotomies have been proposed to address this in revision surgery, with most of the procedures using a supratuberosity or transtuberosity approach. Although satisfactory results have been presented, severe complications involving the extensor mechanism can occur. In this Technical Note, an infratuberosity anterior closing-wedge high tibial osteotomy is demonstrated for slope correction in anterior cruciate ligament–deficient knees in the revision setting.

Anterior cruciate ligament (ACL) injuries continue to be a challenge for orthopaedic surgeons. Despite advancements in surgical techniques, graft failure rates can range from 5.2% to as high as 34.2%; this rate can be even higher in the revision setting.^1^^,^^2^ Recent studies have identified several risk factors for failure that can be classified based on intrinsic and extrinsic factors.^2^^,^^3^ Of note, increased posterior tibial slope (PTS) may increase graft forces and predispose to failure after ACL reconstruction.^4^^,^^5^ Currently, a slope-correcting osteotomy is recommended by some authors in patients that have PTS ≥12° to minimize graft failure.^6^ In current literature, several methods that have been described to achieve slope correction.^2^^,^^7^, ^8^, ^9^, ^10^, ^11^, ^12^, ^13^ Dejour and Bonnin^14^ reported their results of the procedure termed “tibial deflexion osteotomy” and had satisfactory results in the setting of a revision ACL reconstruction. In another study, DePhillipo et al.^11^ reported their technique, which required a tibial tuberosity (TT) osteotomy to complete the correction. Currently, there is limited literature on slope correction with infratuberosity osteotomies. The purpose of this Technical Note is to present an infratuberosity anterior closing-wedge high tibial osteotomy (ACW-HTO) that is performed by the senior author (M.O.).

## Surgical Technique

A comprehensive video of our technique is shown in Video 1. The pearls and advantages of this procedure are shown in Table 1 and Table 2, respectively.

**Table 1:** Pearls and Pitfalls of the Procedure

Pearls	Pitfalls
• Precise planning starting point of osteotomy, which is generally 3 cm below the TT to ensure adequate bone stock remaining to avoid fracture of the TT. • Accurate positioning of fluoroscopy to ensure you can obtain true lateral views of the knee prior to the start of surgery. • Intraoperatively, mark out the osteotomy starting point; confirm osteotomy gap prior to cutting with a ruler and mark with a bovie; this is dependent on your correction size. • Use k-wires to guide your cut, and ensure k-wires are parallel and aimed superior and posterior, targeting the PCL insertion site. • Cool the saw throughout the cutting process with copious saline irrigation. • Use fixation devices (e.g., plate) that allow for compression of the osteotomy through compression holes. • Gap closure should be done in full extension to allow adequate axial compression of the osteotomy site.	• Inadequate preoperative planning • Failure to obtain true lateral fluoroscopic images • Starting point of osteotomy is too close to the TT, risking fracture • Malpositioning of k-wires, leading to poor osteotomy cut and difficult gap closure • Intraoperative hinge fracture, which can be mitigated with additional hinge k-wire

PCL, posterior cruciate ligament; TT, tibial tubercle.

**Table 2:** Advantages and Limitations of Approach

Advantages	Limitations
• Can be performed concomitantly with ACL reconstruction • Reliable method to achieve adequate correction • Can be performed through same incision that is used for hamstring graft harvesting • Direct and safe approach that minimizes chances of severing the neurovascular bundles • Ascending osteotomy cuts allows for relatively easier gap closure than conventional slope-correcting osteotomies	• Diaphyseal osteotomy cuts tend to have higher rates of delayed or nonunion • Need for stronger fixation method (e.g., locked plate extending to diaphysis) to achieve better stability at the osteotomy site • The approach is in proximity to the patellar tendon, which may hinder exposure and placement of retractors; the proximal fragment may also be distracted proximally by the patella tendon

ACL, anterior cruciate ligament.

### Indications and Contraindications

The relative indications for this procedure include failed primary ACL reconstruction with posterior tibial slope ≥12°. Relative contraindications include primary ACL reconstruction in patients with PTS <15°, severe genu varus or valgus deformity, severe hyperextension (recurvatum) of the knee, posterior cruciate ligament deficiency, and severe osteoarthritis of the knee (Kellgren-Lawrence grades 3-4).

### Preoperative Planning

**Fig 1:**
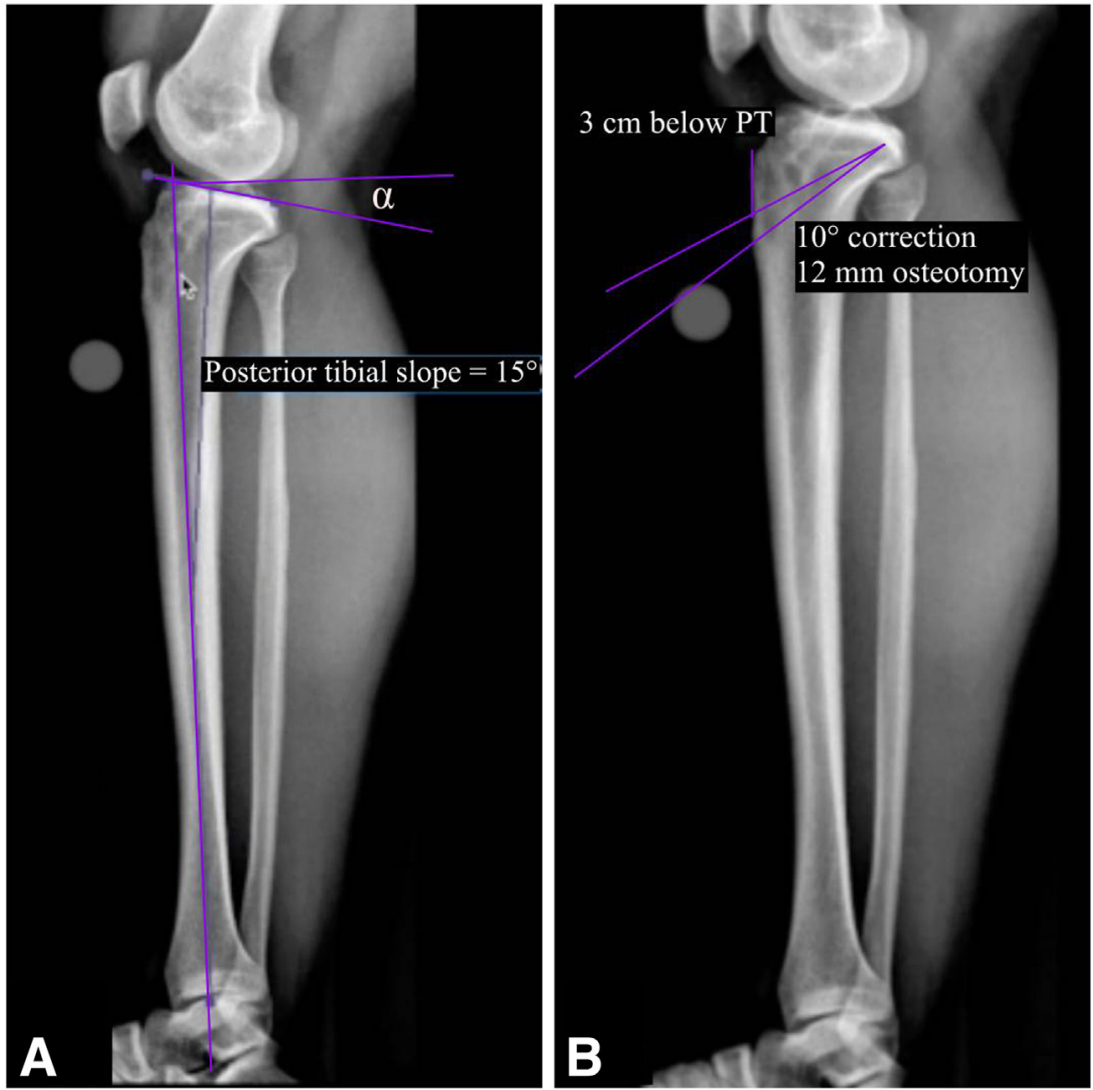
Radiographic measurements (long-leg lateral x-ray views). (A) Measuring posterior tibial slope (α). (B) Determining start point and osteotomy size.

The surgical planning is performed with the lateral full-length tibia radiograph using the PeekMed software. A first line is drawn parallel to the tibial plateau, and a second line is drawn from the most anterior part of the tibial plateau through the center of the tibial metaphysis and diaphysis. The angle formed between these 2 lines is then subtracted from 90° and defined as the posterior tibial slope (Fig 1A). The anticipated correction angle and width (millimeters) of the osteotomy wedge is calculated (Fig 1B). The targeted PTS after surgical correction is recommended to be between 4° and 6°.^15^

### Surgical Approach

**Fig 2:**
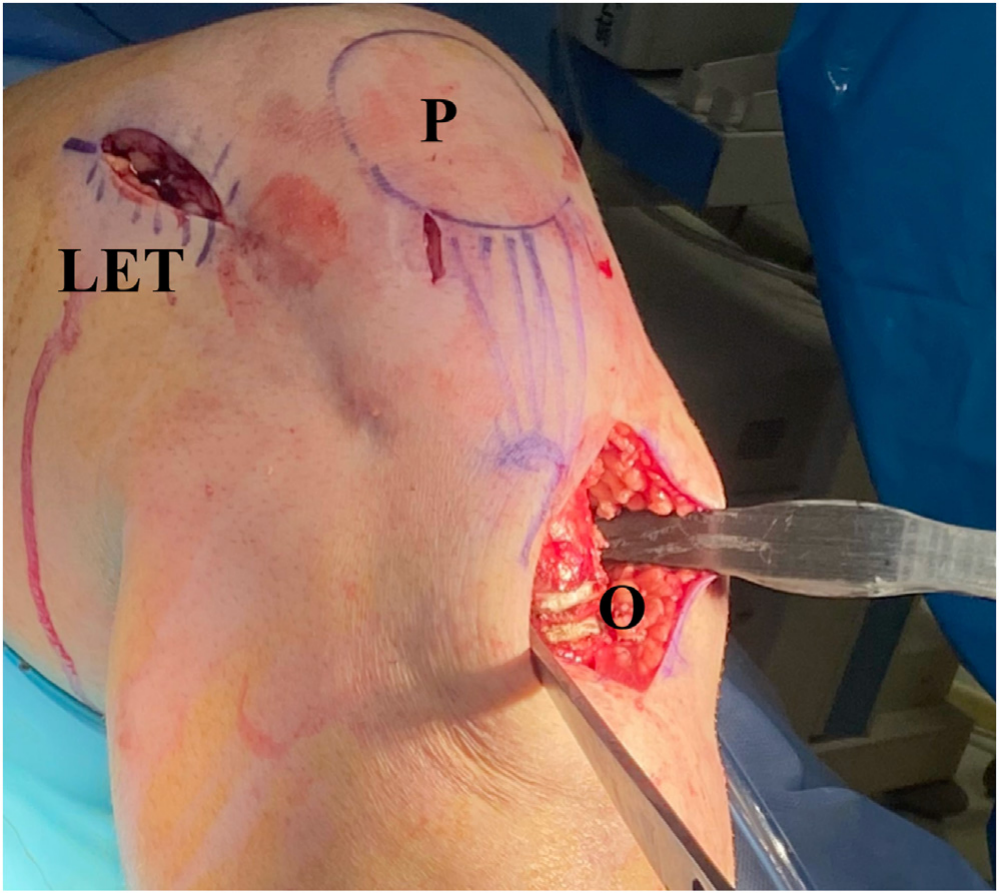
Position of leg and incision (knee flexed in 90°). (LET, lateral extraarticular tenodesis; O, osteotomy site; P, patella.)

**Fig 3:**
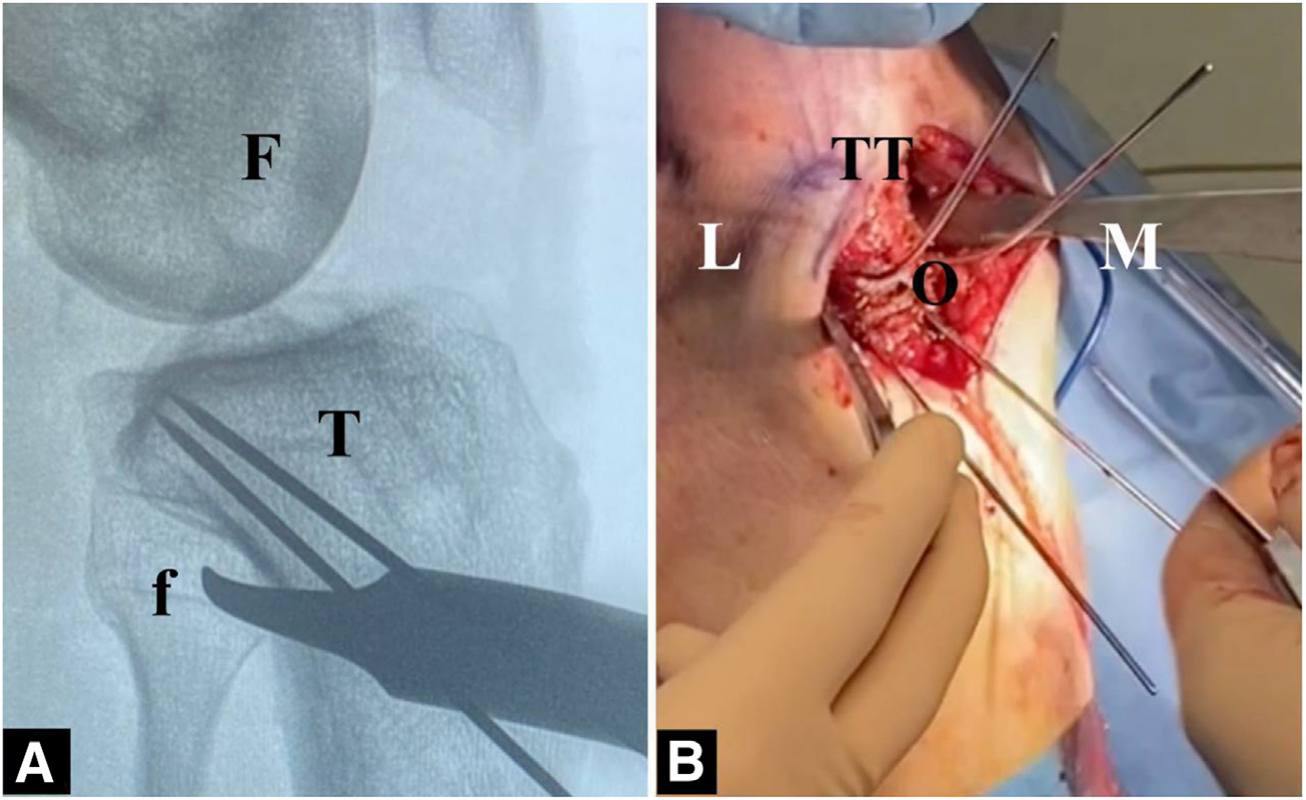
Positioning of k-wires. (A) Fluoroscopy confirming k-wire position. (B) Intraoperative view of k-wires. (F, femur; f, fibula; L, lateral; M, medial; O, osteotomy site; T, tibia; TT, tibial tuberosity.)

**Fig 4:**
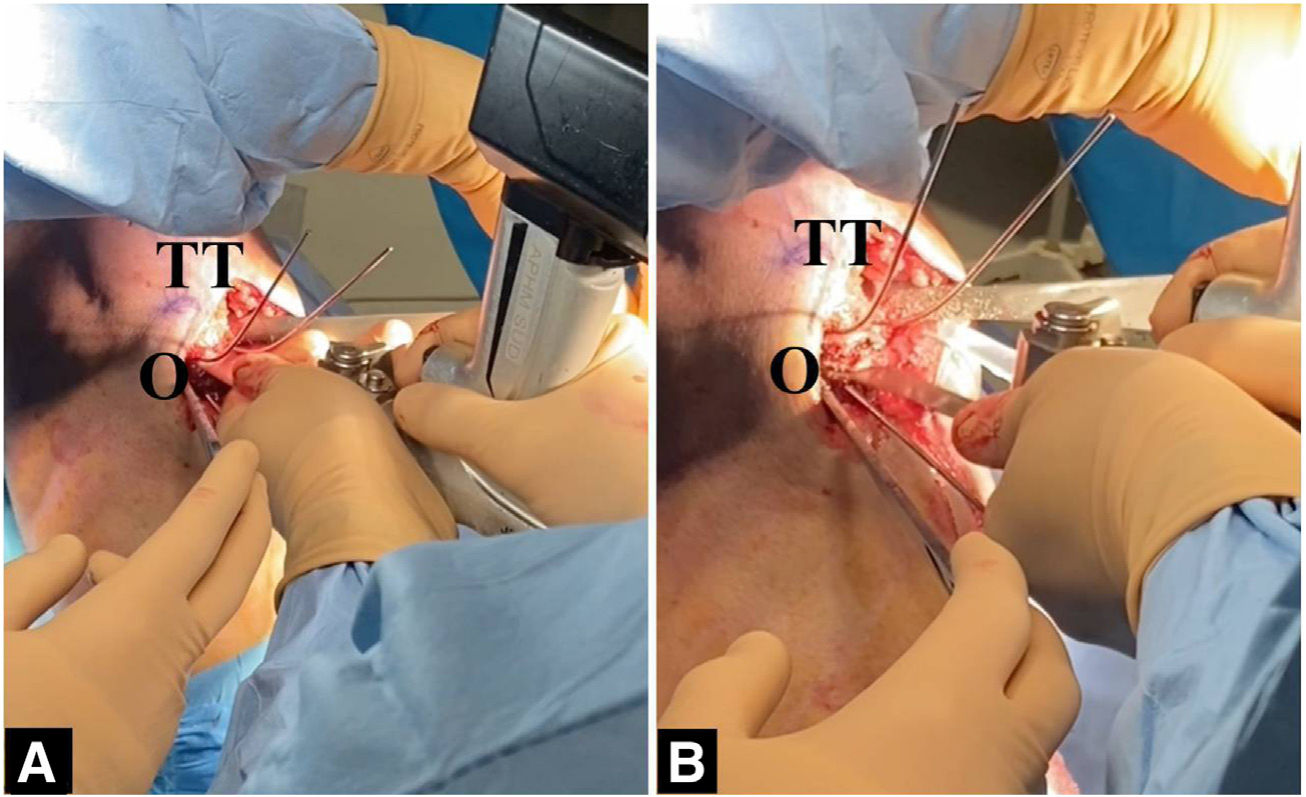
Performing the osteotomy cut. (A) Begin with the superior cut. (B) Inferior osteotomy cut. (O, osteotomy site; TT, tibial tuberosity.)

**Fig 5:**
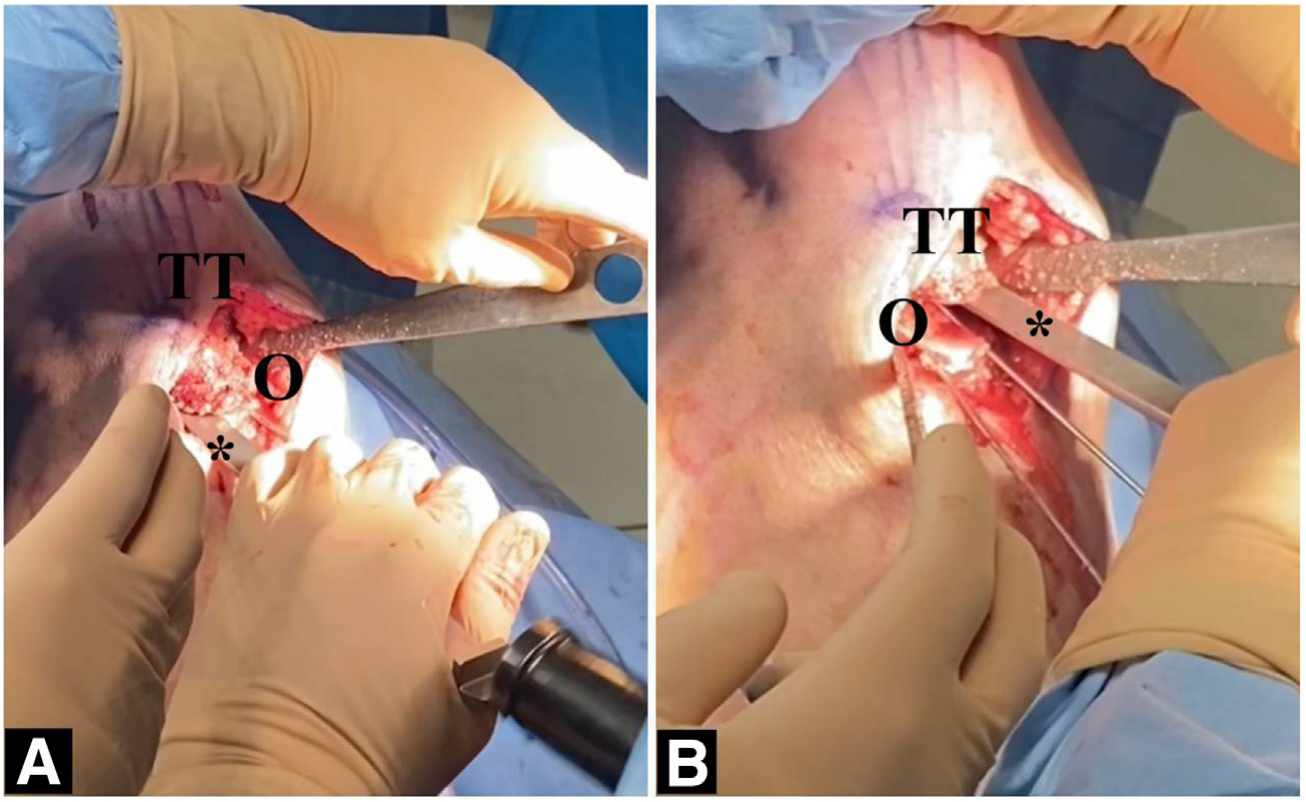
Completing the osteotomy cut. (A) Using an appropriate-sized osteotome to complete the cut. (B) Final osteotomy cut. Asterisk indicates the osteotome. (O, osteotomy site; TT, tibial tuberosity.)

**Fig 6:**
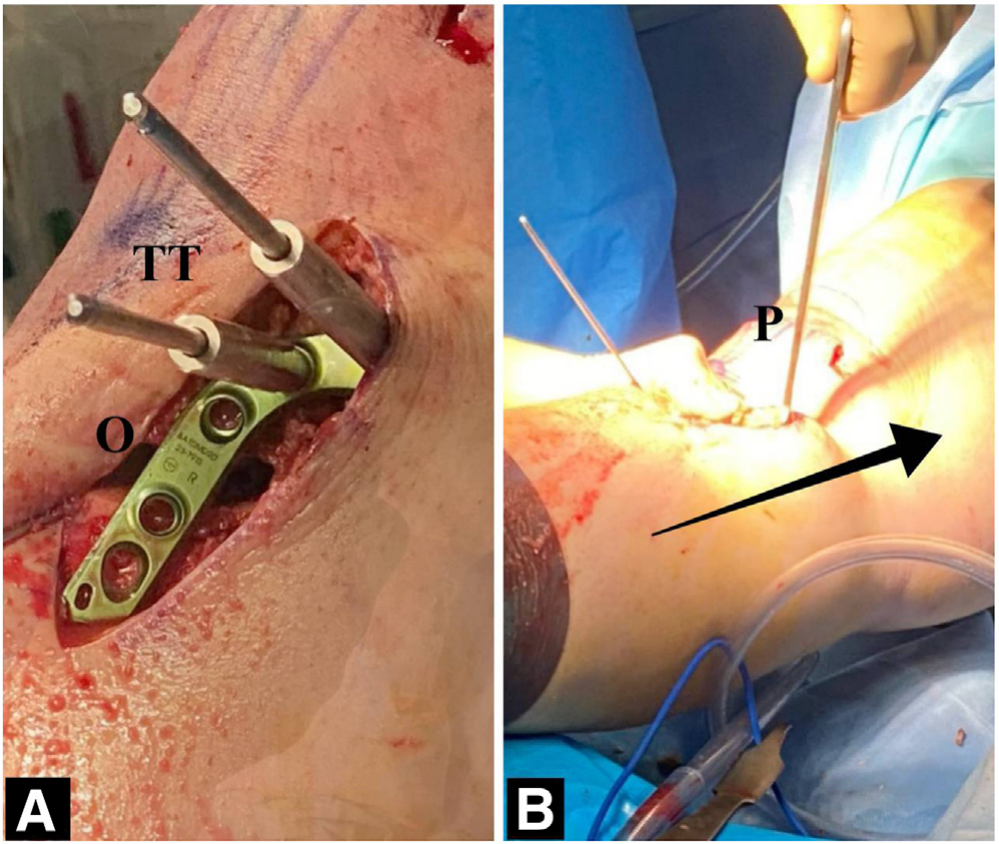
Fixation and osteotomy closure. (A) Position of plate and insertion of the 2 most proximal screws first. (B) Osteotomy gap closure in full extension and inserting the remaining screws, starting with the screw hole located in the osteotomy site to compress the osteotomy. Arrow indicates axial force from inferior to superior. (O, osteotomy site; P, patella; TT, tibial tuberosity.)

The procedure is performed with the patient in the supine position. A well-padded tourniquet is applied in the proximal thigh, and a bump is placed under the ipsilateral hip. A complete diagnostic arthroscopy is first completed. The associated procedures, including revision ACL surgery, meniscal and cartilage procedure, and extra-articular procedures, can be performed prior to the start of the osteotomy (Table 3). Below are the steps involving the slope-correcting osteotomy. Depending on graft choice for the ACL, the incision for hamstring harvesting or patella tendon harvesting can be used for the osteotomy. If a new incision is to be made, this is made over the medial border of the tibia starting from below the TT extending about 5 to 7 cm distally. The sartorial fascia, medial collateral ligament, and the pes anserinus are elevated with a Cobb elevator. Similarly, the Cobb is used to elevate the anterior tibialis muscle and soft tissue around the TT extending to about 3 to 5 cm below the TT. Two Hohmann retractors are placed medially and laterally to expose the surgical site as shown in Figure 2. Electrocautery is then used to mark the superior osteotomy cut (3 cm below the tibial tubercle). Under fluoroscopy, two 1.6-mm k-wires are inserted in a parallel fashion, aiming from anteroinferior to posterosuperior toward the posterior cruciate ligament (PCL) insertion (Fig 3A). Depending on the size of correction, a second line is marked (appropriate number of millimeters to achieve desired correction according to the preoperative planning) below the first mark, indicating the inferior osteotomy cut. Again, two 1.6-mm k-wires are inserted toward the PCL insertion (Fig 3B). Using the 4 k-wires as a guide, 2 ascending osteotomy cuts are made with a hinge point 5 to 10 mm anterior to the PCL insertion site (Fig 4 A and B). A 1-inch osteotome is used carefully to complete the osteotomy cuts (Fig 5 A and B). After a satisfactory osteotomy cut is made, preliminary closure of the osteotomy should be tested at this point to ensure adequate closure prior to fixation. Closure of the osteotomy site should be done in full knee extension, with a compressive axial force directed superiorly from the bottom of the foot. A 6-hole locked-compression plate (Newclip Technics) is applied with the third screw placed directly at the osteotomy site (Fig 6A). After the 2 proximal screws have been inserted, the leg is brought into full extension and compressive axial forces are used to close the osteotomy (Fig 6B). Once satisfactory closure is achieved, a compression screw is drilled and inserted over the third screw hole to secure the osteotomy site. Finally, the 3 distal screws are inserted accordingly, concluding the osteotomy procedure.

**Table 3:** Combined Revision ACL Reconstruction and Infratuberosity ACW-HTO

Steps
1. Diagnostic arthroscopy 2. Preparation of femoral tunnels for ACL and ALL reconstruction 3. Address meniscal pathology (partial meniscectomy vs repair) 4. Perform cartilage procedure as needed (chondroplasty vs cartilage restoration) 5. Perform ACW-HTO 6. Preparation of tibial tunnels for ACL and ALL 7. Fixation of ACL and ALL (double fixation at the tibia)

ACL, anterior cruciate ligament; ACW-HTO, anterior closing-wedge high tibial osteotomy; ALL, anterolateral ligament.

### Postoperative Protocol

**Fig 7:**
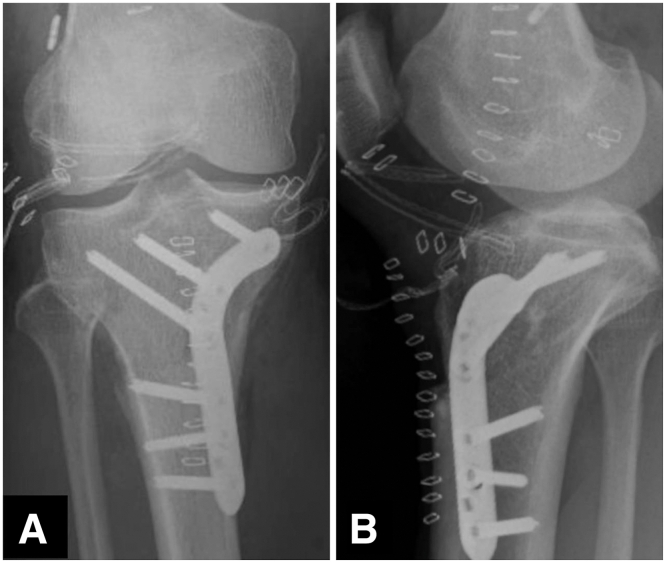
Immediate postoperative radiographs (standard knee x-ray). (A) Anteroposterior view. (B) Lateral view.

After the procedure, the operative extremity is to remain nonweightbearing for 3 weeks on crutches and in a knee immobilizer. Postoperative radiographs are taken in the recovery room to ensure appropriate alignment and hardware position (Fig 7 A and B). Home physical therapy begins on postoperative day 1 with a focus on pain control and reducing swelling. The knee is locked in full extension for the first week. Beginning from postoperative 1 week, progressive passive range of motion from 0° to 90° is tolerated. At 3 weeks, patients can begin progressive weightbearing and range of motion as tolerated. If a 2-stage ACL revision surgery is planned, we recommend waiting for solid union of the osteotomy and complete healing of the bone tunnels.

## Discussion

PTS is one of the main risk factors for failure after ACL reconstruction, and several slope-changing osteotomies have shown promising results.^11^^,^^14^ The most significant changes are a decrease in graft forces, anterior tibial translation, and failure rates in revision ACL surgery.^2^^,^^10^^,^^16^ ACW-HTO can be divided into 3 subtypes (supratuberosity, transtuberosity, and infratuberosity) with respect to the TT. Our institution prefers the infratuberosity approach presented as it offers many technical advantages (Table 2). One of the main advantages is this approach can be effectively performed concomitantly with ACL reconstruction in a single stage. As the osteotomy is performed 3 cm below the tuberosity, it has ample space in the metaphyseal region of the tibia to allow for tunnel preparation. This can also avoid changing the patella heigh, which can be seen in supratuberosity approaches.^17^ In addition, the procedure can be performed by slightly extending the graft harvesting incisions by 1 to 2 cm, effectively reducing the number and length of incision required. Another important advantage is the minimal risk to the neurovascular structures around the knee, as we leave 5 to 10 mm of hinge in the posterior tibia. In addition, a PCL tunnel drill guide can be used to assist for k-wire positioning, which can potentially mitigate risk of damaging the neurovascular structures. Another potential option is to place a blunt Hohmann retractor posteriorly in the proximal tibial to protect the neurovascular structures. Using 1 or a combination of the above steps can effectively avoid violating the neurovascular structures. Meanwhile, there are several mechanical and biological differences with conventional slope-correcting osteotomies. First, closing the osteotomy gap in this procedure can be achieved in full knee extension with a slightly higher tolerance for axial force than the conventional high tibial osteotomy. In our experience, we have not had any intraoperative or postoperative hinge fractures occur in patients who received the infratuberosity ACW-HTO. A recent systematic review also showed no hinge fractures across 5 studies that evaluated patients who underwent ACW-HTO.^18^ Another difference is that the osteotomy cut is made closer to the diaphysis as opposed to conventional proximal tibial osteotomies, in which the cut is made in the metaphysis.^2^^,^^11^^,^^14^ In diaphyseal healing, the bone undergoes both periosteal and medullary callus formation, whereas metaphyseal healing occurs exclusively through medullary callus formation.^19^ Whether this affects union rates in osteotomies remains to be determined. Some of the potential risks and complications specific to this procedure include potential nonunion of the osteotomy site and a posterior hinge fracture. These complications can be mitigated with a stronger fixation device (e.g., locked plate) recommended as opposed to using screws or staples to fix the diaphyseal site and a posterior hinge k-wire, respectively. A potential limitation of this technique is exposure can be challenging as the patella tendon can be in proximity to your osteotomy site. Thorough elevation of the soft tissue around the patella tendon can improve overall visualization. In this Technical Note, we presented our preferred technique, an infratuberosity ACW-HTO in the setting of a failed ACL reconstruction. We believe it is an effective and reproducible approach that can be completed in a 1-stage procedure. Future large cohort studies are required to further elucidate the long-term results of this procedure.

## Disclosures

The authors declare the following financial interests/personal relationships which may be considered as potential competing interests: M.O. is a consultant or advisor for Newclip Technics. W.D. is a consultant or advisor for Newclip Technics and Arthrex. All other authors (K.M.K., S.O., T-F.A.C.) declare that they have no known competing financial interests or personal relationships that could have appeared to influence the work reported in this paper.

## Supplementary Data

Video 1 In this video, we will demonstrate our technique using an infratuberosity anterior closing-wedge high tibial osteotomy for slope correction in anterior cruciate ligament (ACL)–deficient knees. These are our disclosures. We begin with preoperative planning. The 3 key components are measuring the posterior tibial slope, assessing the start point of our osteotomy cut, and determining the osteotomy size depending on our desired correction. The surgical planning is performed with the lateral full-length tibia radiograph using the PeekMed software. Briefly, a first line is drawn parallel to the tibial plateau, and a second tangential line is drawn from the most anterior part of the tibia through the center of the tibial metaphysis and diaphysis. The angle formed between these 2 lines is then subtracted from 90° and defined as the posterior tibial slope (Fig 1A). The anticipated correction angle and width (millimeters) of the osteotomy wedge is calculated (Fig 1B). We always start 3 cm below the patella tendon insertion with our hinge positioned about 5 mm anterior to the posterior cruciate ligament (PCL) insertion site. In this patient, a 10° correction corresponds to a 12-mm cut. The targeted posterior tibial slope after surgical correction is recommended to be between 5° and 8°, as prior studies have raised concerns of overcorrection leading to increased strain on the PCL. We then switch to the surgical procedures. Several steps have been taken before the osteotomy, including graft harvesting and preparation, arthroscopic exploration, meniscus and cartilage repair, femoral tunnel for the ACL, and the lateral tenodesis. The approach is slightly medial, extending the classic hamstring harvesting approach. We then elevate the medial collateral ligament and protect it with an Hohmann retractor. We do the same for the anterior tibialis muscle, incising first the periosteum and aponeurosis of the muscle, with a Hohmann retractor used, protecting it from the beginning to the end. We mark the distal aspect of the tibial tuberosity, 3 cm below the insertion of the patella tendon. The x-ray on the lateral view needs to be perfect with complete superimposition of the 2 condyles. We then shoot 2 ascending k-wires, positioned distally onto the tibial tuberosity. This will help us drive the saw blade ascending from this anterior point to the insertion of the PCL. That is the hinge point of the osteotomy. This fragment is resected, which can be measured to confirm the length is around our desired cut. The osteotomy is then completed by cutting the remaining part of the triangle, on the lateral and medial side. The plate is positioned anteriorly, once we feel the osteotomy is moving. With the knee in flexion, the position of the plate needs to be perfect, which can be confirmed with x-rays. Then we shoot the 2 proximal screws to close the osteotomy with compressive screws. We verify the position of those 2 screws with respect to the joint line and extend the leg to compressive osteotomy with recurvatum measures. We use a compressive screw to compress the osteotomy site onto the bone and finish the locking holes with 3 locking screws. We always verify the position of the plate. The next step will be the tibial tunnel, ACL graft fixation that needs to be doubled on the tibia, fixation of the lateral tenodesis, and finally skin closures. These are the final x-rays of the patient, in frontal and sagittal views with complete closure of the osteotomy gap. Postoperative rehabilitation includes nonweightbearing for 3 weeks. Thank you for watching.
